# A Specific High Toxicity of Xinjunan (Dioctyldiethylenetriamine) to *Xanthomonas* by Affecting the Iron Metabolism

**DOI:** 10.1128/spectrum.04382-22

**Published:** 2023-03-06

**Authors:** Hao-Yu Zang, Xue Yang, Chun-Yan Gu, Jia-Zhi Sun, Rui Pan, Yong-Xing Wang, Tong-Chun Gao, Shan-Kui Yuan, Yu Chen

**Affiliations:** a Institute of Plant Protection and Agro-Products Safety, Anhui Academy of Agricultural Sciences, Hefei, China; b School of Plant Protection, Anhui Agricultural University, Hefei, China; c Shandong Vicome Greenland Chemical Co. Ltd., Jinan, China; d Institute for the Control of Agrochemicals, Ministry of Agriculture and Rural Affairs, Beijing, China; e Key Laboratory of Integrated Crop Pest Management of Anhui Province, School of Plant Protection, Anhui Agricultural University, Hefei, China; f Engineering Laboratory for Green Pesticide Development and Application of Anhui Province, School of Plant Protection, Anhui Agricultural University, Hefei, China; g Key Laboratory of Biology and Sustainable Management of Plant Diseases and Pests of Anhui Higher Education Institutes, School of Plant Protection, Anhui Agricultural University, Hefei, China; Institute of Plant Protection, Chinese Academy of Agricultural Sciences

**Keywords:** Xinjunan (Dioctyldiethylenetriamine), *Xanthomonas*, bactericide, iron uptake, mode of action

## Abstract

*Xanthomonas* spp. encompass a wide range of phytopathogens that brings great economic losses to various crops. Rational use of pesticides is one of the effective means to control the diseases. Xinjunan (Dioctyldiethylenetriamine) is structurally unrelated to traditional bactericides, and is used to control fungal, bacterial, and viral diseases with their unknown mode of actions. Here, we found that Xinjunan had a specific high toxicity toward *Xanthomonas* spp., especially to the Xanthomonas oryzae pv. *oryzae* (*Xoo*), the causal agent of rice bacterial leaf blight. Transmission electron microscope (TEM) confirmed its bactericidal effect by morphological changes, including cytoplasmic vacuolation and cell wall degradation. DNA synthesis was significantly inhibited, and the inhibitory effect enhanced with the increase of the chemical concentration. However, the synthesis of protein and EPS was not affected. RNA-seq revealed differentially expressed genes (DEGs) particularly enriched in iron uptake, which was subsequently confirmed by siderophore detection, intracellular Fe content and iron-uptake related genes transcriptional level. The laser confocal scanning microscopy and growth curve monitoring of the cell viability in response to different Fe condition proved that the Xinjunan activity relied on the addition of iron. Taken together, we speculated that Xinjunan exerted bactericidal effect by affecting cellular iron metabolism as a novel mode of action.

**IMPORTANCE** Sustainable chemical control for rice bacterial leaf blight caused by Xanthomonas oryzae pv. *oryzae* need to be developed due to limited bactericides with high efficiency, low cost, and low toxicity in China. This present study verified a broad-spectrum fungicide named Xinjunan possessing a specific high toxicity to *Xanthomonas* pathogens, which were further confirmed by affecting the cellular iron metabolism of *Xoo* as a novel mode of action. These findings will contribute to the application of the compound in the field control of *Xanthomonas* spp.-caused diseases, and be directive for future development of novel specific drugs for the control of severe bacterial diseases based on this novel mode of action.

## INTRODUCTION

*Xanthomonas* is a large genus of Gram-negative, rod-shaped, yellow-pigmented bacteria that are closely associated with plants (1). The majority of *Xanthomonas* species are phytopathogens that cause serious diseases to more than 400 host plants, including a wide variety of economically important crops, such as rice, citrus, banana, cabbage, tomato, pepper, and bean, with individual members usually exhibiting a high degree of host specificity (2, 3) According to the vote of plant bacteriologists in 2012, three species of this genus (i.e., Xanthomonas oryzae, Xanthomonas campestris, and Xanthomonas axonopodis), were selected as the “Top 10” plant-pathogenic bacteria based on scientific/economic importance (4). Pathogens of this genus cause critical economic losses worldwide (5, 6); therefore, it is vital to search for effective prevention and control methods for the diseases caused by this genus.

Bacterial leaf blight (BLB), caused by Xanthomonas oryzae pv. *oryzae* (*Xoo*), is found in both tropical and temperate regions, making 20% to 50% yield loss according to the annual disease development (4, 7). BLB can be controlled by the use of resistant rice cultivars. However, this resistance is usually overcome by *Xoo* due to its capacity to express various effectors (8). Chemical control is still one of the main methods to control the disease. At present, copper compounds, antibiotics, and thiazole compounds are most widely used to control BLB in China (9). Nevertheless, the shortcomings such as poor efficacy, high phytotoxicity, and bactericide resistance (10–13) appear gradually, compelling the researchers to find alternative bactericides against BLB in China and other rice producing regions.

Dioctyldiethylenetriamine (IUPAC name: N^1^-octyl-N^2^-[2-(octylamino)ethyl] ethane-1,2-diamine, CAS No. 57413-95-3, also known as Xinjunan in China; Fig. S1) is a lipophilic large molecule with weak water solubility, medium polarity, pyrolysis, and no obvious UV absorption (14). This chemical is a broad-spectrum fungicide widely used on many crops, including vegetables and fruits in China (15, 16). It was first reported for agriculture use in 1990 as a disinfectant and sterilant protecting plants from viral and fungal infection. Dioctyldiethylenetriamine and its analogues were also used in sanitation purposes since the 1990s (17). Chinese common name ‘‘Xinjunan’’ is approved for Dioctyldiethylenetriamine acetate by the Pesticide Standardization Administration of the People’s Republic of China and is mostly formulated as 1.8% AS (18). Since 1991, Xinjunan has been registered for use in a variety of crops in China, including apple, pepper, tomato, cotton, and rice (16). As China's early policy did not require the disclosure of toxicological data of the pesticide, so, the toxicity of Xinjunan to different types of organisms is very limited. Only one research article (14) reported that Xinjunan has a high toxicity to Zebrafish, and a strong bioconcentration, which might be concentrated through the food chain step by step and engender a potential risk to ecological environment. However, according to the manufacturer's report (unpublished data), the acute toxicity of this chemical to male rats and female rats is low (5,843.4 mg/kg.b.wt and 4,300 mg/kg.b.wt, respectively). Field trials showed that Xinjunan disappeared rapidly in the rice plants and fields under natural conditions with the half-lives range 3.9 to 6.6 and 6.2 to 12 days, respectively (18). Based on the supervised residue trials and the risk assessment results, MRL for Xinjunan in rice was recommended at 0.1 mg/kg in China. The intakes of Xinjunan residue on rice is far less than ADI (<0.2%), indicating that daily consumption of rice products when applied with Xinjunan under the GAP use pattern do not cause potential health risk (18).

Xinjunan has been applied for many years in China, and its chemical structure is quite different from that of previous pesticides but its mode of action is relatively less studied. In contrast, the mode of actions of the current main bactericides used to control *Xanthomonas*-caused diseases were well studied. For instance, streptomycin seems to involve in distorting the ribosome so that transition from initiation of the complex (30S-mRNA-tRNA) to chain elongating ribosome is blocked. Thus, the normal sequence of translation is disrupted, the bacteria is unable to synthesize proteins vital for its cell growth, and thereby fails to survive (19). Besides, Streptomycin also affects bacterial cells by impairing translation of mRNA, leading to the production of defective proteins (20). Copper-based bactericides are toxic to cells mainly due to its interaction with nucleic acids, disruption of enzyme active sites, interference with the energy transport system, and ultimately, the disruption of the integrity of cell membranes (21–23). Bismerthiazol, a thiadiazole molecule, does not greatly inhibit *Xoo* growth *in vitro*, but reduces disease by inhibiting the histidine utilization pathway and quorum sensing (24). Nevertheless, solidifying pathogen’s protein, destroying the pathogen’s cell membrane and inhibiting pathogen’s respiration was earlier reported as mode of action of Xinjunan (25), but the specific physiochemical mechanism is still not clear, and these effects may not be the initial action sites. On the other hand, *in vitro* experiment showed that the toxicity of Xinjunan to different types of microorganisms varied greatly, suggesting that there were significant differences in the mode of action on different microorganisms (16). Thus, it is necessary to clarify the mode of action of Xinjunan on specific microorganisms for its effective use.

The aims of this study were: (i) to determine the toxicity of Xinjunan against *Xanthomonas* species and compare with other plant pathogens; (ii) investigate the physiochemical changes of *Xanthomonas* at cellular and molecular levels in response to Xinjunan treatment; and (iii) to study the specific mode of action of Xinjunan on *Xanthomonas* spp.

## RESULTS

### Xinjunan exhibited specific high toxicity against *Xanthomonas* spp.

Nineteen strains, including plant-pathogenic fungi, bacteria, and plant growth-promoting rhizobacteria (PGPR), were selected to evaluate the antimicrobial activity of Xinjunan. The effective concentration for 50% inhibition (EC_50_) of the chemical on each strain was measured as a reduction in cell density or colony diameter after incubation. EC_50_ values of Xinjunan to 19 strains differed from 0.33 to 62.66 μg/mL, as shown in Table 1. Xinjunan showed the strongest effect against *Xanthomonas* spp. with EC_50_ ranging from 0.33 to 0.80 μg/mL. This specific high toxicity suggests that Xinjunan may have a unique mode of action to *Xanthomonas* spp. Meanwhile, MIC of Xinjunan against *Xanthomonas* spp. was also determined by agar dilution method as shown in Table 2. Xinjunan showed bactericidal effect against *X. oryzae* pv. *oryzae* (*Xoo*) strains with an MIC of 16 μg/mL, which was significantly lower than others. Furthermore, we tested the toxicity of Xinjunan on 10 different Xanthomonas oryzae strains. The EC_50_ values varied from 0.33 to 5.03 μg/mL and the average EC_50_ was 0.996 μg/mL (Table S1). Thus, we took *Xoo* as a model for the following studies.

**TABLE 1:** Determination of Xinjunan EC_50_ for 19 different strains

Strains	EC_50_ (mg/L)	Regression equation	*R* ^2^
Pathogenic fungi			
Fusarium graminearum	49.00	Y = 2.5271 + 1.4627X	0.99
Sclerotinia sclerotiorum	18.09	Y = 1.9285 + 2.4426X	0.98
*Colletotrichum gloeosporioides*	18.69	Y = 2.2424 + 2.1694X	0.99
*Clonostachys rosea*	42.75	Y = 1.0945 + 2.3946X	0.97
*Botryosphaeria dothidea*	62.66	Y = 1.4775 + 1.9602X	0.98
*Lasiodiplodia theobromae*	33.34	Y=-1.0465 + 3.9701X	0.99
*Fomitiporia torreyae*	35.45	Y = 1.6693 + 2.1494X	0.98
Fusarium sp.	39.43	Y = 1.7487 + 2.0374X	0.98
*Alternaria Mali*	37.39	Y = 2.3697 + 1.6724X	0.99
Pathogenic bacteria			
Xanthomonas oryzae pv. *oryzae*	0.33	Y = 9.4995 + 9.359X	0.94
Xanthomonas oryzae pv. *oryzicola*	0.53	Y = 6.5044 + 5.446X	0.99
Xanthomonas campestris pv. *campestris*	0.80	Y = 2.285 + 5.1599X	0.99
Pectobacterium carotovorum subsp. *carotovorum*	7.12	Y = 3.5412_+1.7116X	0.98
Pseudomonas syringae pv. *lachrymans*	6.17	Y = 4.1725 + 1.0467X	0.97
Pseudomonas syringae pv. *actinidiae*	29.71	Y = 3.4752X-0.1188	0.97
Acidovorax citrulli	6.93	Y = 4.1540 + 1.0061X	0.99
PGPRs			
Paenibacillus polymyxa	15.57	Y = 3.2673 + 2.324X	0.93
Bacillus pumilus	12.41	Y = 2.9144 + 1.9065X	0.99
Bacillus subtilis	12.76	Y = 4.0456 + 2.1675X	0.91

**TABLE 2:** MIC determination of Xinjunan against *Xanthomonas* spp.

Strains	Dilution fold^*a*^	Xinjunan concentration (mg/L)								
		0	0.5	1	2	4	8	16	32	64
Xanthomonas oryzae pv. oryzae PX099^A^	×1	+++	+++	+++	+++	++	+	−	−	−
	×3	+++	+++	+++	++	+	−	−	−	−
	×9	+++	+++	+++	++	−	−	−	−	−
Xanthomonas oryzae pv. oryzicola RS105	×1	+++	+++	+++	+++	++	++	+	−	−
	×3	+++	+++	+++	+++	++	+	+	−	−
	×9	+++	+++	+++	+++	+	+	−	−	−
Xanthomonas campestris pv. campestris WT8004	×1	+++	+++	+++	+++	++	++	++	+	−
	×3	+++	+++	+++	+++	++	++	+	−	−
	×9	+++	+++	+++	+++	++	+	+	−	−

a 1×, 3×, and 9×: bacterial suspensions in late exponential growth phase were diluted at different multiples.

### Effect of Xinjunan on *Xoo* cell morphology.

**FIG 1:**
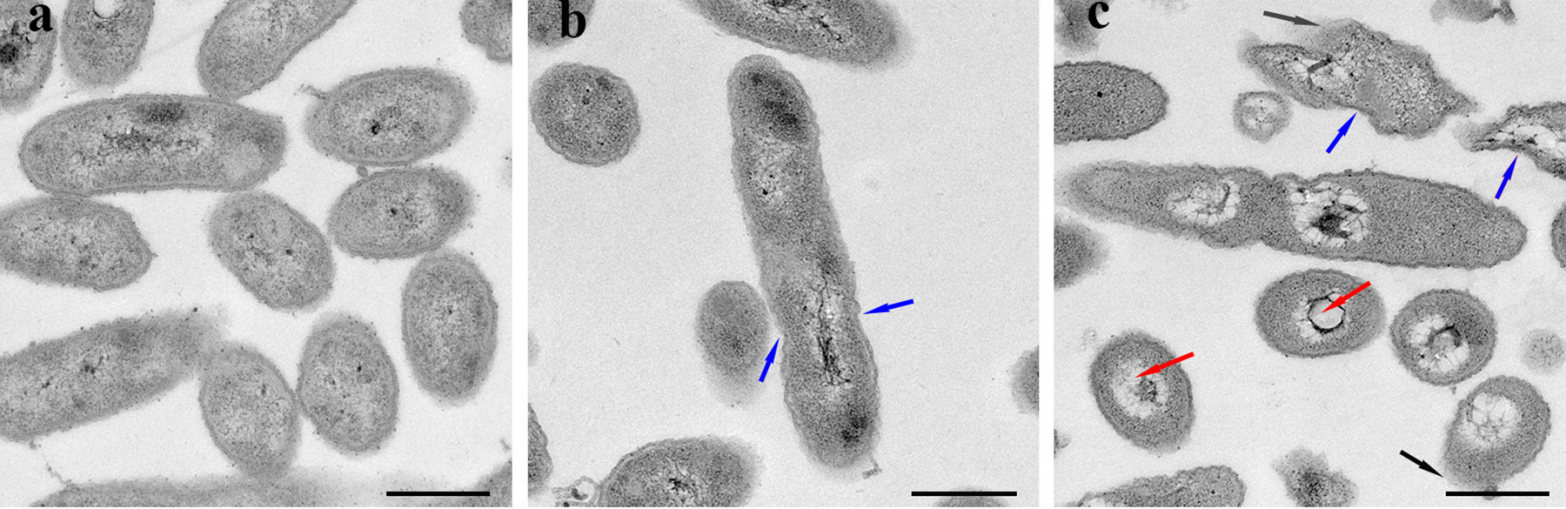
Effects of Xinjunan on cell structure of *Xoo*. (a to c) Transmission electron microscopy images. (a) Negative-control cells treated with 1% DMF. (b) Cells treated with Xinjunan at 1/2 MIC for 30 min. (c) Cells treated with Xinjunan at 1/2 MIC for 4 h. Black arrows indicated cell wall degradation, blue arrows indicated cell wall sunken or corrugated, red arrows indicated cytoplasmic vacuolation. Scale bars represent 500 nm.

The growth curve was determined by adding a series of Xinjunan with concentration gradients. The growth rate of *Xoo* was completely inhibited while culturing in media with Xinjunan at MIC. Xinjunan had no obvious influences on the growth of *Xoo* at early stage (0.5 h) at the concentrations of 1/32, 1/16, 1/8, 1/4, and 1/2 MIC. Two hours later, each treatment began to exhibit the difference gradually (Fig. S2). Based on these results, Xinjunan with a concentration of 1/4 MIC and 0.5 h/4 h were selected as treatment condition for cell structure observation. Transmission electron microscope (TEM) was carried out to evaluate the level of cell wall damage and intracellular modification in *Xoo* PXO99^A^ treated by Xinjunan. *Xoo* cells exposed to 1% dimethylformamide (DMF) served as the negative control, which showed an intact outline of cell wall and a peptidoglycan layer (Fig. 1a). *Xoo* cells treated with Xinjunan at 1/4 MIC for 0.5 h were partial sunken or corrugated (Fig. 1b), and these situations were further exacerbated after treated for 4 h. In addition, cytoplasmic vacuolation and cell wall degradation can be found in some cells after 4 h (Fig. 1c). TEM results confirmed the bactericidal activity of the chemical, and suggested that it should take a certain time for Xinjunan to produce a marked effect.

### Xinjunan inhibited DNA synthesis of *Xoo*.

**FIG 2:**
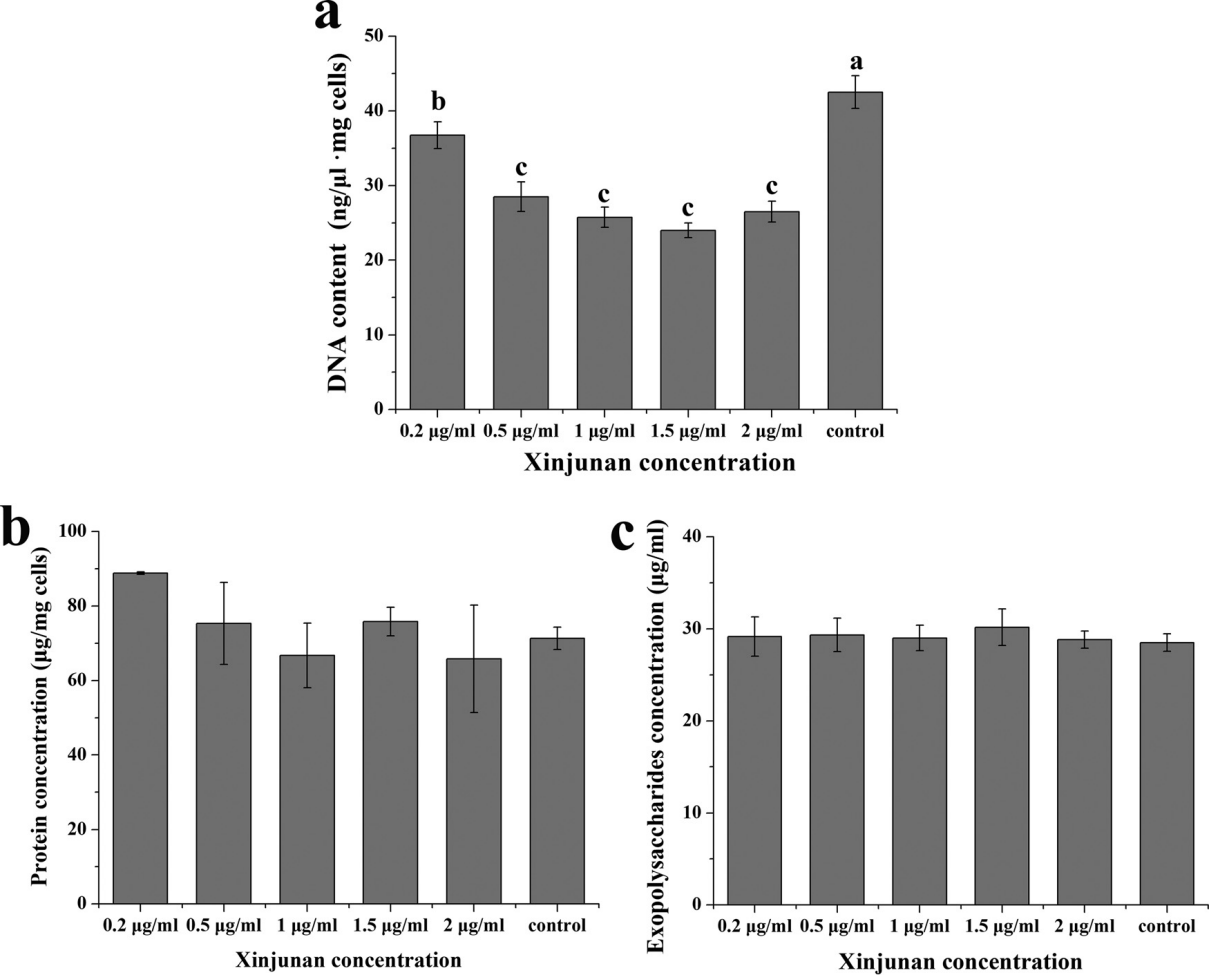
Effect of Xinjunan on the synthesis of macromolecules of *Xoo*. (a) DNA content; (b) protein content; (c) EPS content. Bars represent the standard deviation (*n* = 3). *, *P* < 0.05.

In order to study the effect of Xinjunan on macromolecules synthesis of *Xoo*, different concentrations of Xinjunan were set up to investigate the DNA/protein/exopolysaccharides (EPS) contents variation in *Xoo* cells. As shown in Fig. 2, Xinjunan has a significant inhibitory effect on DNA synthesis of *Xoo*. Under 0.2 μg/mL treatment, the inhibition rate of DNA synthesis was 13.5%, and in a certain range of concentration, the inhibition effect improved with the concentration of agent increasing. In the same condition, Xinjunan had little effect on the synthesis of extracellular polysaccharide or protein, indicating that the drug may exert a specific action on the *Xoo* cells by inhibiting the biosynthesis of bacterial DNA.

### Effect of Xinjunan on Fe homeostasis of *Xoo*.

**FIG 3:**
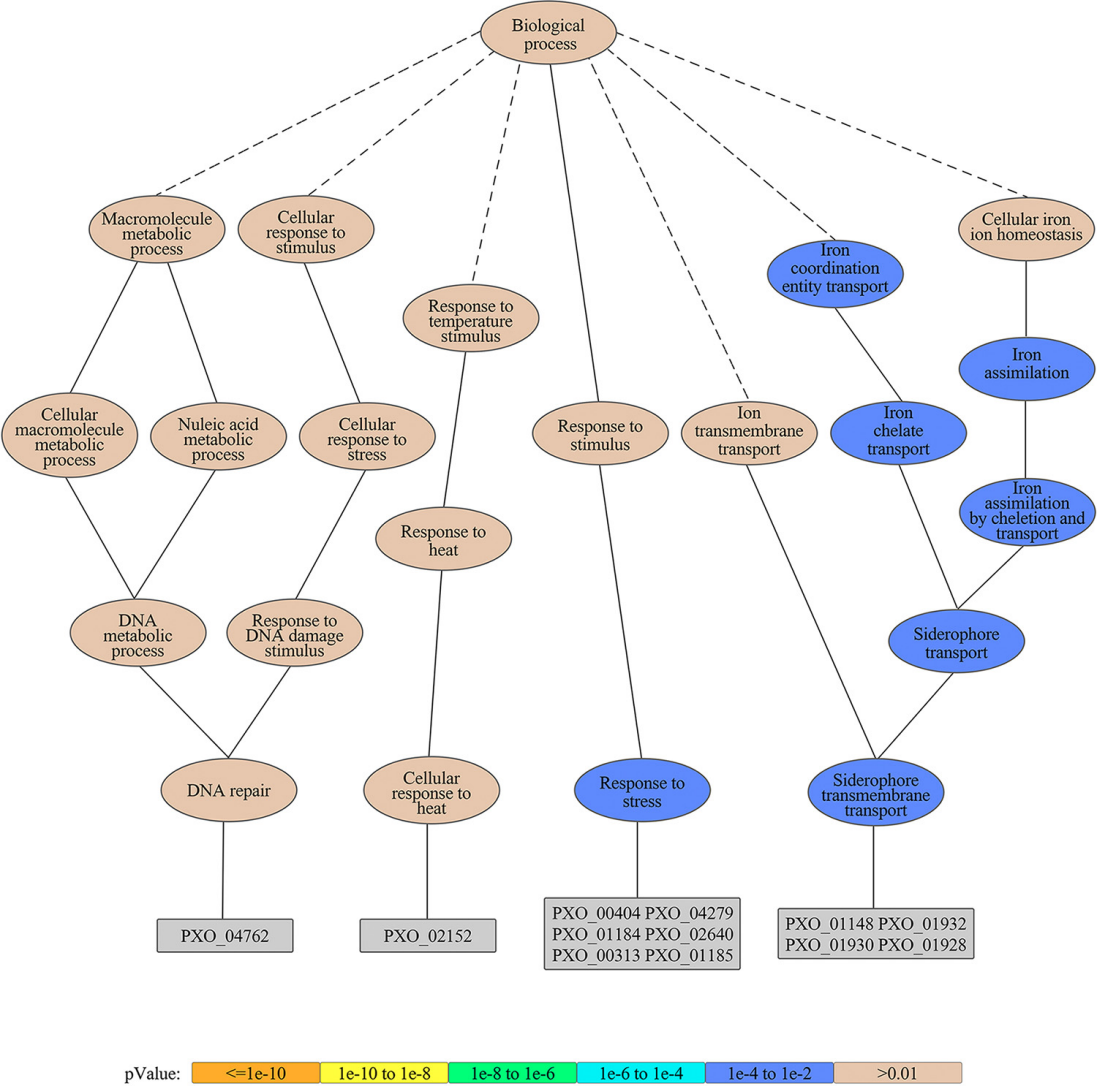
The gene ontology (GO) enrichment analysis revealed DEGs involved in iron uptake. GO terms with corrected *P* value less than 0.01 were considered significantly enriched by differential expressed genes.

**FIG 4:**
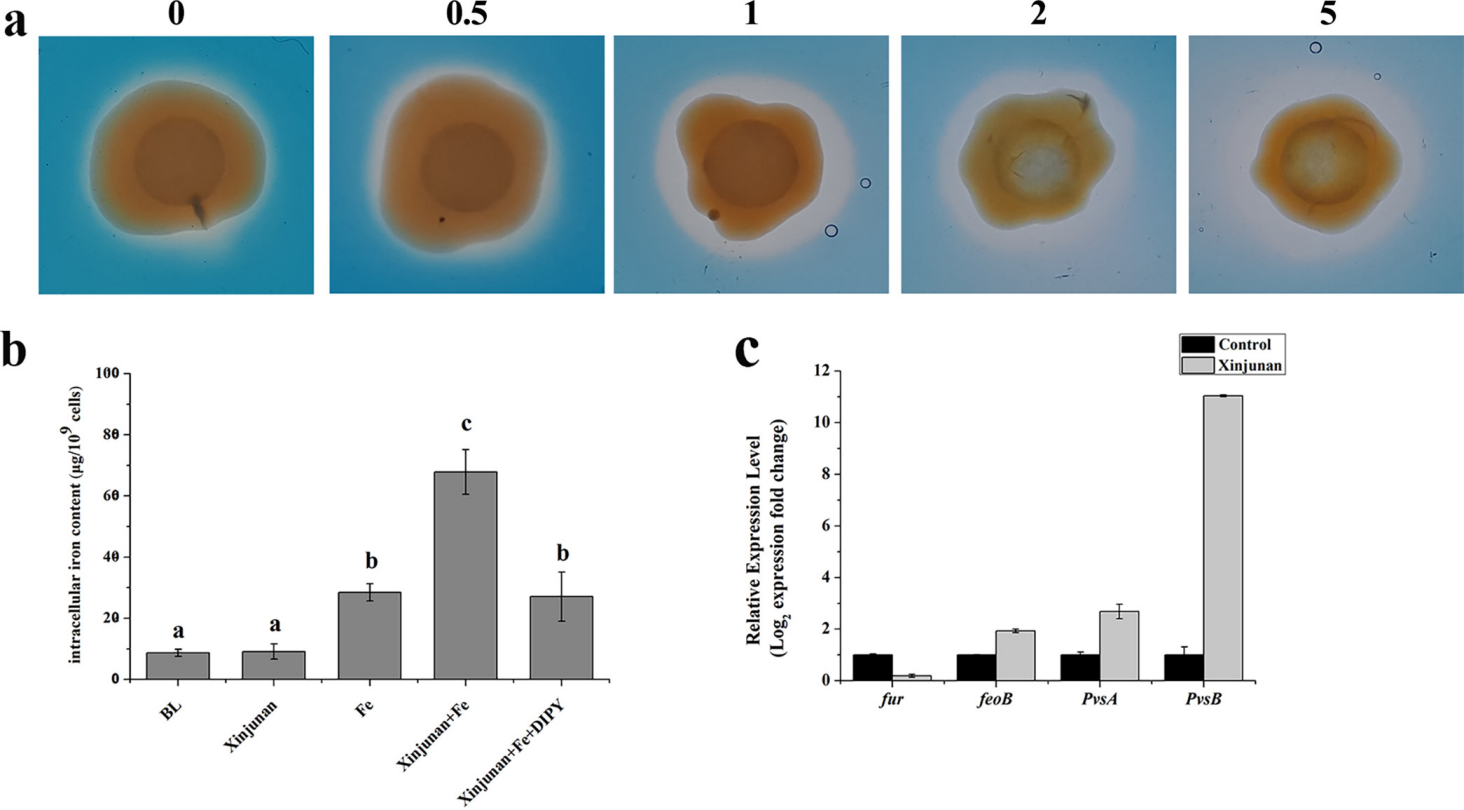
Xinjunan affect Fe homeostasis of *Xoo*. (a) Siderophore production in response to different concentrations of Xinjunan, by using the Chrome azural S (CAS) assay to detect siderophore secretion, which appeared as a yellow halo zone. (b) Intracellular iron content of *Xoo* in iron-depleted or iron-replete conditions, the iron atom content was measured by ICP-OES. Iron concentration was calculated by dividing the total iron atom value by the number of bacterial cells. BL, blank control containing only MMX medium; Xinjunan, MMX medium plus 4 μg/mL Xinjunan; Fe, MMX medium plus 100 μM FeCl_3_; Xinjunan+Fe, MMX medium plus 4 μg/mL Xinjunan and 100 μM FeCl_3_; Xinjunan+Fe+DIPY, MMX medium plus 4 μg/mL Xinjunan, 100 μM FeCl_3_, and 125 μM dipyridyl. (c) The relative expression levels of iron-uptake related genes. The *FeoB*, *pvsA*, *pvsB*, and *fur* transcriptional level were determined by real time RT-PCR.

Based on the above findings, we aimed to figure out the target genes that might be interfered by Xinjunan. We first adopted RNA-seq to determine the overall situation of transcriptional changes under the Xinjunan treatment. Overall, 56 of 4,346 genes were differentially expressed, including 53 upregulated and three downregulated genes. A KEGG-pathway analysis revealed the differentially expressed genes (DEGs) were mainly involved in metabolic pathway (13.89%), two-component system (8.33%), and microbial metabolism in diverse environments (16.67%) (Fig. S3 to 4). The gene ontology (GO) enrichment analysis was applied to describe characteristics and reaction network of DEGs. GO terms with corrected *P* value less than 0.01 were considered significantly enriched by differential expressed genes. The results showed that the DEGs enriched not only in response to stress responses, but also, in particularly, involved in iron uptake (Fig. 3). To verify these results, we subsequently detected the siderophore production using a Chrome azural S (CAS) agar plate. Results showed that when Xinjunan was added *in vitro*, the siderophore produced by *Xanthomonas* was significantly increased, and the degree of increase was positively correlated with the concentration of this chemical (Fig. 4a). Meanwhile, we measured the intracellular iron content of *Xoo* in iron-depleted or iron-replete conditions using inductively coupled plasma optical emission spectrometry (ICP-OES). After growth for 3 h in the iron-depleted MMX medium, the iron accumulation in cells of *Xoo* strains remained the same level either treated with Xinjunan or not. However, in the iron-replete medium (MMX + 100 μM FeCl_3_), the iron concentration in the *Xoo* were significantly increased by 2.38-fold under Xinjunan treatment (Fig. 4b). When additional dipyridyl (DIPY), the specific chelating agent for iron ions, was added into the medium, the intracellular iron level immediately reduced to the same level as treated with Fe alone. Further investigation regarding iron-uptaking genes transcription level was also determined by quantitative PCR. *FeoB*, *pvsA*, and *pvsB* were involved in *Xanthomonas* iron transportation and siderophore synthesis, whereas *fur* plays negative regulatory role on iron uptake. Real-time RT-PCR analysis of these genes revealed that *feoB*, *pvsA*, and *pvsB* expression levels were higher, whereas *fur* expression was downregulated compared with control (Fig. 4c). Taken together, these results supported the view that Xinjunan could affect Fe homeostasis of *Xoo* by disrupting bacterial iron uptake.

### Xinjunan activity is associated with environmental Fe content.

**FIG 5:**
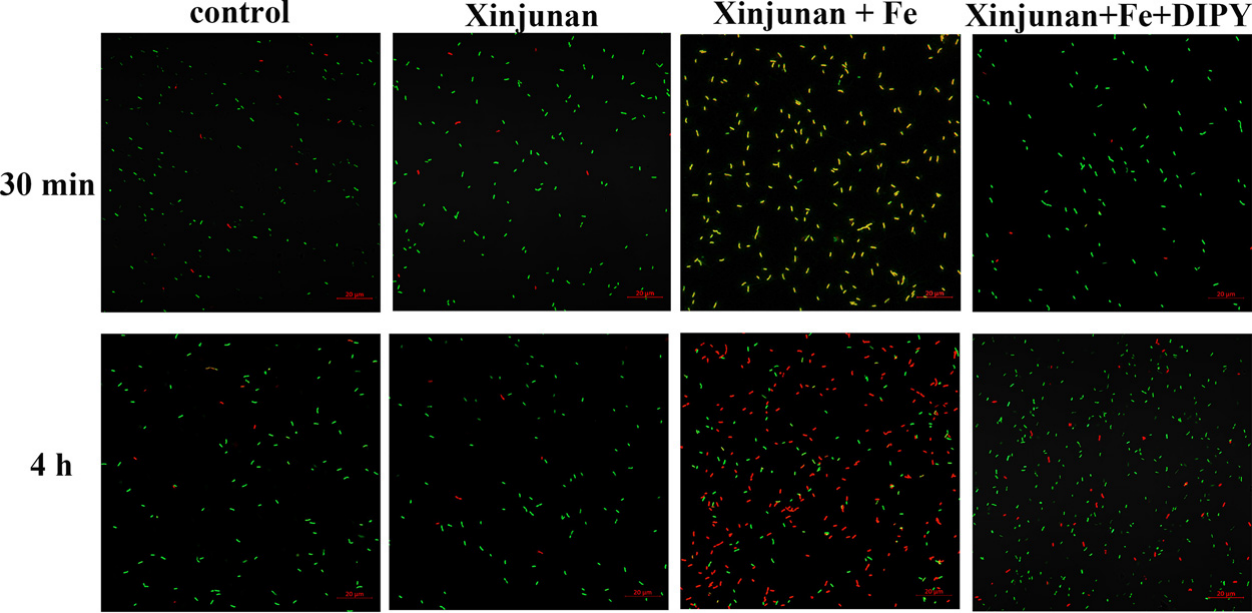
Cell viability in response to Xinjunan under different Fe conditions. Pictures were taken using Confocal laser scanning microscopy photographs. Bacteria with intact cell membranes stain fluorescent green, whereas bacteria with damaged membranes stain fluorescent red. Control, MMX plus 100 μM FeCl_3_; Xinjunan, MMX plus Xinjunan at concentration of 1/4 MIC; Xinjunan+Fe, MMX plus Xinjunan (1/4 MIC) and 100 μM FeCl_3_; Xinjunan +Fe+ DIPY, MMX plus Xinjunan (1/4 MIC), 100 μM FeCl_3_, and 125 μM iron chelating agent DIPY. Imaged cells with filters listed below: Ex/Em 480/550 for SYTO9, Ex/Em 490/635 for PI.

**FIG 6:**
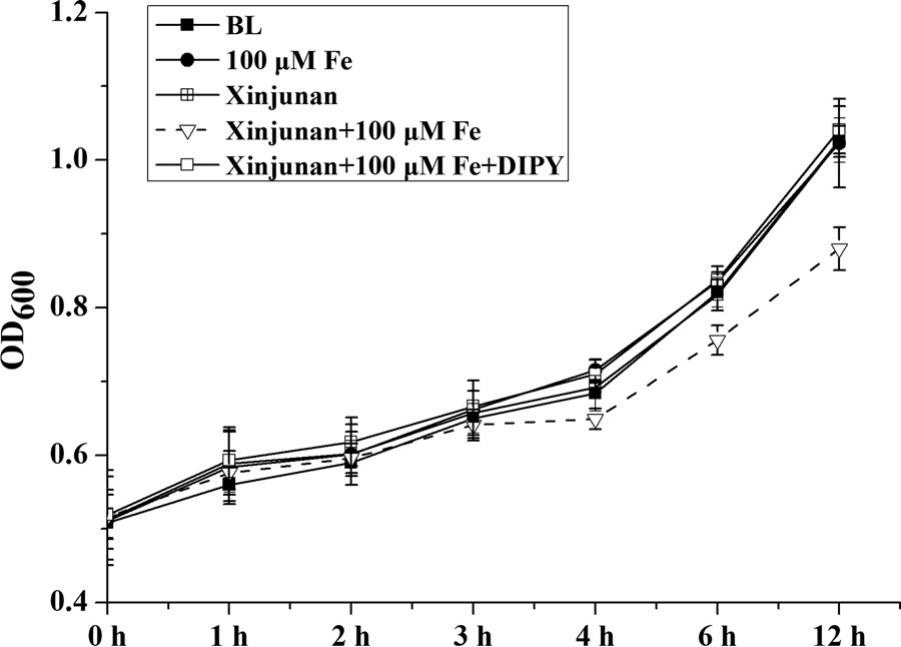
*Xoo* growth curve in response to Xinjunan under different Fe conditions. *Xoo* was initially cultured in NB liquid medium with continuous shaking at 28°C until exponential growth phase. The *Xoo* cells were harvested by centrifugation and washed three times with PBS buffer. *Xoo* cells were then transferred into fresh MMX medium with a final OD adjusted to of approximately 0.5. BL, add DMF solvent as negative control; 100 μM Fe, add additional Fe^3+^ to a final concentration of 100 μM; Xinjunan, only add Xinjunan to a final concentration of 1 mg/L; Xinjunan + 100 μM Fe, simultaneously add 100 μM Fe^3+^ and 1 mg/L Xinjunan; Xinjunan + 100μM Fe + DIPY, simultaneously add Fe^3+^/Xinjunan and 150 μM iron chelating agent DIPY. Each value represents the average of three independent measurements. Bars represent the standard deviation.

To further confirm the role of Fe content in Xinjunan anti-bacterial activity, we adopted confocal laser scanning microscopy to visualize the cell viability in response to Xinjunan treatment with or without Fe. Bacteria with intact cell membranes stain fluorescent green, whereas bacteria with damaged membranes stain fluorescent red. When *Xoo* were solely treated with Xinjunan, the cells exhibited the same level of green fluorescence compared to mock (Fig. 5) in both 0.5 h and 3 h treatment. However, when added 100 μM FeCl_3_ together with Xinjunan, the majority of bacteria cells became yellow-red after 0.5 h treatment, and completely turned into red “dead” cells after 3 h. When additional DIPY, the specific chelating agent for iron ions, was introduced, the *Xoo* cells remained green for the whole time. In addition, we also investigated the growth curve in response to different Fe condition. The results were consistent with the cell viability observation (Fig. 6). When Xinjunan was added alone, there was little inhibition compared with the control. However, when additional Fe^3+^ was added, an obvious inhibitory effect could be observed, and when DIPY was added simultaneously, the inhibitory effect disappeared. Thus, we concluded that the environmental Fe content is necessary for the bactericidal activity of Xinjunan.

## DISCUSSION

Chemical control is still an important tool for BLB management due to the lack of stable resistant cultivars against the disease in China. A bacterial blight resistance analysis of China National authorized rice varieties showed that only 78 varieties (4.3%) showed disease resistance among the 1,805 tested varieties in a regional trial, accounting for 4.3% (26). Bismerthiazol had been the most commonly used bactericide for controlling BLB in China since 1970s; however, bismerthiazol-resistant *Xoo* has been reported at various fields in different regions of China (10, 27–29). The use of some other bactericides like copper agent and agricultural streptomycin are recently forbidden or restricted in China. As a result, the effective BLB controlling agents are very few in the market. Therefore, introduction of novel bactericides for sustainable BLB control is recommended.

Despite Xinjunan being applied in agricultural use for almost 30 years in China, it is mainly used to control fungal diseases, especially for fruit trees diseases (16, 30). Meanwhile, little is known about the mode of action of Xinjunan on different types of pathogens. In this study, Xinjunan seems to be more effective against bacterial pathogens, indicating that Xinjunan may be a good candidate for controlling bacterial diseases. The results revealed that *Xanthomonas* spp., among all tested pathogens, were particularly sensitive to this chemical. Considering the differences in structure and chemical properties compared to other commonly used bactericides, it was suggested that the Xinjunan might have a unique mode of action against *Xanthomonas* and is safe to the nontarget organisms. On the other hand, it might be difficult to produce cross-resistance with previous bactericides. In addition, this compound can be synthesized from diethylenetriamine and bromoalkane in one step with a yield high up to 87.6% (31), which indicated that this bactericide has great advantages in manufacturing process and is cost-effective for sustainable control of BLB or other bacterial diseases.

The current study confirmed the addition of Xinjunan-caused morphological changes in *Xoo* cells, mainly the cell wall structure damage, resulting in leakage of cellular components and cytoplasmic vacuolation. The results showed that the compound had bactericidal effect rather than bacteriostatic effect. However, this kind of damage seems to take certain time, and the degree is not as strong as other reported drugs. For instance, terpenoids target the cytoplasmic membrane, which could disrupt lipid–protein interaction, increase membrane permeability, and ultimately destroy cell integrity (32). Exposure to diffcidin or bacilysin makes cell walls loose and porous, distorting from their normal shape or even be ruptured (33). In connection with the latter results that the Xinjunan did not damage the cell integrity without iron or with extra DIPY addition, the quantitative detection results showed that the chemical could inhibit *Xoo* nucleic acid synthesis. It is reasonable to believe that Xinjunan may not directly damage and interact with the cell wall structure of *Xoo* but interfere with the related pathways of cell wall synthesis by affecting the expression of specific genes.

RNA-seq of *Xoo* in response to Xinjunan treatment offered us a global view to seek potential mode of action. As showed by GO enrichment analysis, the DEGs are interestingly involved in iron uptake terms. Iron is vital in the progress of life; excessive or insufficient intake of iron will both affect cell metabolism (34, 35). This was consistent with the results that Xinjunan inhibited *Xoo* nucleic acid synthesis.

**FIG 7:**
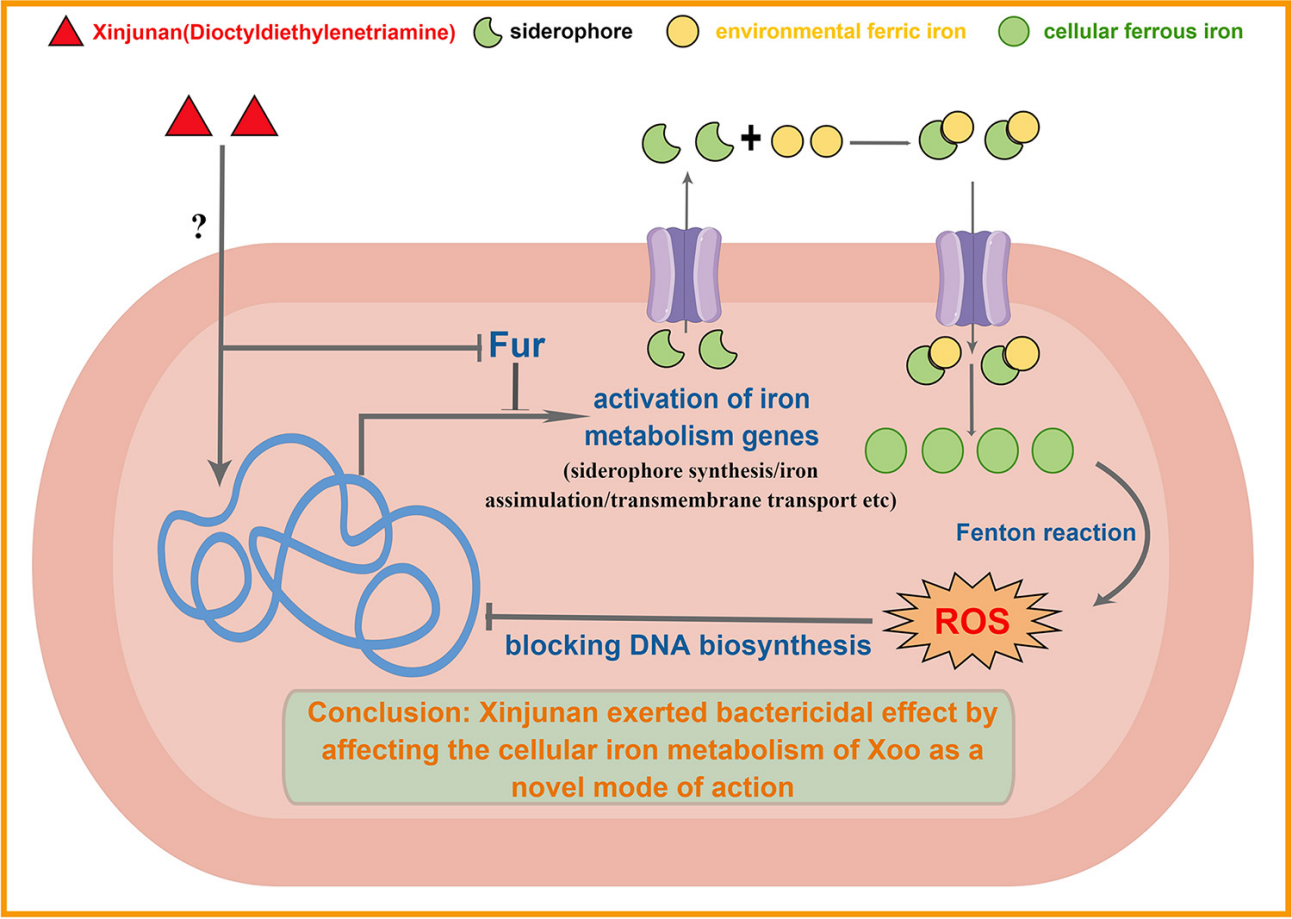
Schematic diagram of the novel mode of action underlying the specific high toxicity of Xinjunan against *Xanthomonas*.

Based on the above findings, we sequentially verified the effect of Xinjunan on cell growth and viability under strict control of iron environment. LCSM images of *Xoo* cells intuitively appeared almost entirely green in the untreated and sole addition of Xinjunan groups, indicating most cells remained alive and intact under iron-depleted condition. By contrast, cells exposed to Xinjunan together with Fe for 4 h would lead to red dead cells. Additionally, when iron chelating agent was added, almost all *Xoo* cells remained green, implying that the bactericidal activity was closely related with Fe content. This meaningful discovery will guide the scientific use of this chemical, and furthermore, will provide a possibility of compound synergism. In summary, the specific high bactericidal effect of Xinjunan on *Xoo* was achieved by affecting the cellular iron metabolism (Fig. 7). When *Xoo* is treated with Xinjunan, a large amount of environmental Fe^3+^ entered and accumulated in the cells, which may convert into Fe^2+^ and destroy the intracellular iron homeostasis. It was reported when the concentration of Fe^2+^ in cells is too high, ferrous ions catalyze Fenton reaction to produce hydroxyl radicals with high activity, which destroy DNA and lead to the cell death. To our knowledge, this was a novel mode of action that has not been recorded in the fungicide resistance action committee (FRAC). Further exploration of the action site/target of the chemical will not only be beneficial to consolidate the research foundation of action mechanism of fungicides, but also be directive for future development of novel specific drugs to control the diseases caused by *Xanthomonas* species.

### Conclusion.

In this research, we proved Xinjunan seems to be more effective against bacterial pathogens, especially to *Xanthomonas* spp., indicating there might be unique target site(s) of this bactericide. We investigated the physiochemical changes of *Xanthomonas* at cellular and molecular levels in response to Xinjunan treatment, which made up a deficiency in related field, and suggested Xinjunan may not directly damage the cell structure but interfere with the related pathways by affecting the expression of specific genes. The mode of action of Xinjunan was investigated for the first time, which confirmed the specific high bactericidal effect of Xinjunan on *Xoo* was achieved by affecting the cellular iron metabolism. This is not only the first time to establish the specific high toxicity/physiochemical effect of Xinjunan to *Xanthomonas* pathogens, but also the first time to reveal a novel mode of action of this unique bactericide.

## MATERIALS AND METHODS

### Reagents, strains, and culture conditions.

In this study, 95% Xinjunan (Dioctyldiethylenetriamine) C_20_H_45_N_3_, CAS No. 57413-95-3 was provided by Shandong Vicome Greenland Chemical Co., Ltd. Fungal and bacterial strains used in this study were stored in our lab. Generally, fungal strains were cultured in PDA medium at 28°C. Bacterial strains were cultured in NB medium with shaking (180 rpm). For Fe detection or cell viability experiments, *Xoo* strains were inoculated into MMX media after enrichment culture. CAS medium was prepared by adding 10 mL of CAS solution to 100 mL of Silva Buddenhagen (SB) agar.

### EC_50_ determination.

For fungal pathogens, the EC_50_ were determined *in vitro* by transferring plugs (5 mm in diameter) of mycelium from the leading edge of an actively growing colony to a series of PDA plates containing different concentration of Xinjunan. PDA medium containing DMF (dimethylformamide), the solvent of Xinjunan, was used as control. The diameters (minus the diameter of the inoculation plug) of the colonies were measured after incubation for 2 to 4 days at 25°C in darkness. The growth inhibition as percentage of control was calculated. The median effective Xinjunan concentration (EC_50_) for the isolates were calculated based on linear regression of colony diameter on log-transformed fungicide concentration. For each concentration, three replications were conducted.

For bacterial strains, the EC_50_ values were determined on the basis of growth inhibition, as described earlier (29). A quantity of 50 μL of the bacterial suspension was added to 25 mL of NB medium in 50 mL Erlenmeyer flasks containing diluted concentrations of Xinjunan. NB medium containing DMF (dimethylformamide), the solvent of Xinjunan, was used as control. The optical density of the suspensions in all flasks was measured when the optical density of the suspension in the control flask increased to about 10^8^ CFU mL^−1^. The log of percentage inhibition based on optical density was regressed on the log of compound concentration, and EC_50_ values were calculated.

### MIC determination.

NA agar medium was mixed with Xinjunan in the culture dish with the final concentration of 0 to 64 mg/L. The bacteria cells were cultured in a flask containing NB medium until late exponential growth stage. Then, 2 μL of the tested bacterial suspensions with different dilution fold (1×, 3×, and 9×) was spotted on the media and incubated at 28°C for further 48 h. NA medium containing DMF was used as control. The MIC is defined as the lowest antimicrobial concentration of Xinjunan that prevents visible growth of the organism investigated after approximately 48 h of incubation.

### Growth curves.

The growth curves were established as previously described with minor modifications (36). Briefly, *Xoo* was cultured in NB liquid medium with continuous shaking at 28°C until exponential growth phase. The culture with fresh NB medium was adjusted to a final OD of approximately 0.4. Xinjunan was added to each well to yield final concentrations of EC_50_ to 1/16 MIC,1/8 MIC, 1/4 MIC, 1/2 MIC, and 1 MIC, while NB containing 1% DMF (vol/vol) was utilized as a negative control. Subsequently, samples were incubated at 28°C under constant shaking, and cell growth was assessed by measuring the optical density at 600 nm from 0 h to 12 h using a microplate reader (Biotek, Winooski, VT).

### TEM analysis.

TEM observation was performed as described by Yoshioka-Nishimura et al. (37) with some modifications. TEM was applied to examine the structural changes occurring in *Xoo* cells treated with Xinjunan. *Xoo* was cultured at the late exponential phase in 20 mL of NB at 28°C, and the cells were sedimented by centrifugation at 4°C (6,000 g, 10 min) and resuspended in PBS buffer to achieve an OD_600_ of 0.4. The cell suspension was treated with Xinjunan at 1/4 MIC, and further cultured for 0.5 h and 4 h at 28°C. The treated and untreated cells were separately sedimented by centrifugation at 4°C (8,000 g, 5 min) and washed three times with PBS buffer. These cells were then fixed with 2.5% (vol/vol) glutaraldehyde overnight at 4°C and were further dehydrated through a graded series of alcohol (30%, 50%, 70%, 90%, and 100%), for 15 min. Thin sections were cut using an ultramicrotome (MT-X; RMC, Jefferson, OH, USA) and subjected to a TEM analysis at an operating voltage of 80 kV.

### Determination of DNA content.

A total of 1 mL exponential-phase *Xoo* liquid culture was inoculated to 25 mL NB broth, then Xinjunan was added to make its final concentrations to 0.2 μg/mL, 0.5 μg/mL, 1 μg/mL, 1.5 μg/mL, 2 μg/mL, respectively. Then, 24 h later, 2 mL of the bacteria were centrifuged and dyed at 50°C. DNA of the above samples was extracted using bacterial genome extraction kit (Omega Bio-Tek, Norcross, GA, USA). The DNA contents were determined by Nanodrop Spectrophotometer.

### Determination of protein content.

*Xoo* were treated with 0.2 μg/mL, 0.5 μg/mL, 1 μg/mL, 1.5 μg/mL, and 2 μg/mL Xinjunan, respectively, as we mentioned above. The liquid culture of each treatment was centrifuged at 12,000 rpm for 5 min to collect the cell pellets. The sediments were washed with normal saline for three times, and then the suspensions were adjusted to the same turbidity. A total of 2 mL of normal saline was added into 4-m suspension of each treatment, prior to ultrasonic disruption (3 min, 3 S crushing, 3 S interval), and thereafter centrifuged at 10,000 rpm for 30 min. The protein concentration (mg/mL) was measured using the Bradford protein assay kit (Beyotime Biotechnology, Shanghai, China), and bovine serum albumin was used as a reference (38).

### Determination of EPS content.

The polysaccharide content was determined by sulfuric acid-phenol method. Briefly, *Xoo* were treated with 0.2 μg/mL, 0.5 μg/mL, 1 μg/mL, 1.5 μg/mL, and 2 μg/mL Xinjunan, respectively, as we mentioned above. A total of 20 mL of bacterial broth was centrifuged at 5,000 rpm for 15 min to collect the supernatant. Three volumes of 95% ice-ethanol (–20°C) were added and mixed thoroughly, and the samples were then left overnight at 4°C. The supernatant was discarded after centrifugation and precipitation was resuspended with 8 mL sterilized water. Next, 1 mL suspension was rapidly mixed with 1 mL of 5% phenol; then, 5 mL of concentrated sulfuric acid was added for a 10 min incubation. The OD_488_ value was determined on a spectrophotometer after water bath at 25°C for 15 min. Glucose gradient solutions ranging from 25 to 200 μg/mL were chosen for standard curve determination.

### RNA-seq and data analysis.

Exponential-phase *Xoo* liquid culture was inoculated to 25 mL NB broth with continuous shaking at 28°C until the OD reached 0.4. Xinjunan was added to make the final concentration of EC_50_ (i.e., 0.33 μg/mL) and continued shaking for 0.5 h. *Xoo* cells were collected by centrifuging at 4°C and washed with PBS buffer twice. DMF was used as negative control. The samples were frozen in liquid nitrogen immediately and sent to BGI company for RNA extraction and cDNA library construction. Later, QC test samples were sequenced via Illumina HiSeq 2000.

The clean reads were obtained by filtration and then aligned to the reference sequence with SOAP aligner/SOAP2 (39). After the comparison, the distribution and coverage of reads on the reference sequence were counted to determine whether the comparison results passed the second quality control.

Then, a series of follow-up analysis, such as gene expression level, prediction of new transcripts, annotation, and SNP detection, were carried out on the data. The DEGs among the samples were screened out from the results of gene expression. The GO enrichment analysis was applied to describe product characteristics and reaction network of DEGs. All DEGs were mapped to GO terms in the database (40, 41). GO terms with corrected *P* value less than 0.01 were considered significantly enriched by differential expressed genes.

### qRT-PCR analysis.

Total RNA was extracted using Omega Bacterial RNA Kit (R6950-01). The reverse transcription reaction was performed using AMV First Strand cDNA Synthesis Kit (Sangon Biotech, B532445-0020) according to the manufacturer’s instructions. The specific primer pairs for *FeoB*, *pvsA*, *pvsB*, *fur*, and *16S* rRNA genes were used as listed in Table S2. The 16S rRNA gene was used as the reference gene. Real-time PCR was performed using SGExcel FastSYBR Master (Sangon Biotech, B532955-0005). The PCRs were run on CFX96 connect real-time PCR system (Bio-Rad, Hercules, CA).

### Siderophore detection.

Siderophore production was determined using a Chrome azural S (CAS) agar plate (42, 43). CAS medium was prepared by adding 10 mL of CAS solution to 100 mL of SB agar (44). Bacterial colonies were spotted onto a CAS plate and incubated aerobically at 28°C for 2 days. The siderophore production was indicated by the presence of a yellow halo zone around the bacterial colony that was seen readily against the blue-green media background. This halo was formed upon the release of a yellowish CAS dye upon competitive binding of the bacterial siderophore with Fe^3+^ from the blue-green CAS-Fe^3+^ complexes (43).

### Bacterial cell viability determination.

Cell viability was determined by LIVE/DEAD BacLight Bacterial Viability Kits L7012 (Molecular Probes, Invitrogen) according to the manufacturer’s instruction. Briefly, *Xoo* cells were grown to an OD_600_ of 1.0 in NYG medium. Cells were collected by centrifugation at 10,000 rpm for 10 min and washed three times with fresh MMX medium. The bacteria were then inoculated into fresh MMX plus 100 μM FeCl_3_ (control), Xinjunan (1/4 MIC), Xinjunan (1/4 MIC) plus 100 μM FeCl_3_, Xinjunan plus 100 μM FeCl_3_ and 150 μM DIPY for 30 min and 4 h. Then, the cells were collected by centrifugation at 5,000 rpm at 4°C for 10 min and the pellets were washed and resuspended in 10 mL of PBS buffer.

Next, 3 μL of the dye mixture (combined equal volumes of SYTO9 and PI) was added to each milliliter of the bacteria suspension and incubated at RT in dark for 15 min. A total of 5 μL of the stained bacterial suspension were put onto a glass slide and covered with a coverslip. Imaged cells with filters listed below: Ex/Em 480/550 for SYTO9, Ex/Em 490/635 for PI.

### Determination of cellular iron concentration.

Cellular iron levels in different strains were determined by inductively coupled plasma-optical emission spectroscopy ICAP 6300 (ICP-OES, Thermo Fisher Scientific, MA USA) as previously described (45). The bacterial cells were grown to an OD_600_ of 1.0 in NB medium. Cells were collected by centrifugation at 8,000 rpm and washed three times with fresh MMX medium. The bacteria were then inoculated into fresh MMX plus 100 μM FeCl_3_/Xinjunan/Xinjunan media (OD_600_ = 0.6). Three hours later, cells were harvested by centrifugation and washed three times with sterilized PBS buffer (KH_2_PO_4_ 0.27 g/L, Na_2_HPO_4_ 1.42 g/L, NaCl 8g/L, KCl 0.2 g/L, pH 7.4). The pelleted cells were oven-dried at 65°C for 72 h and digested with HNO_3_–HClO_4_ (4:1, vol/vol). The digestions were transferred to 25 mL volumetric flasks and the final volumes were made up to 25 mL with H_2_O. The iron atom contents were measured by ICP-OES. The samples not treated by HNO_3_-HClO_4_ (4:1) were used as parallel controls. The total iron concentration for each sample was calculated by dividing the iron atom value by the number of bacterial cells.

### Data analysis.

All analyses were conducted using SPSS 19.0 (Statistical Package for the Social Science, SPSS Inc., Chicago, IL). When ANOVAs were significant (*P* = 0.05), means were separated with Fisher’s least significant difference (LSD).
