# Supplementary material for: A Specific High Toxicity of Xinjunan (Dioctyldiethylenetriamine) to Xanthomonas by Affecting the Iron Metabolism
Source: Microbiol Spectr. 2023 Mar 6;11(2):e04382-22. doi: 10.1128/spectrum.04382-22 (PMC10100656; doi:10.1128/spectrum.04382-22)
Supplement: Supplemental file 1 — Supplemental material. Download spectrum.04382-22-s0001.pdf, PDF file, 0.3 MB [file spectrum.04382-22-s0001.pdf]

Supplementary Materials

A specific high toxicity of Xinjunan (Dioctyldiethylenetriamine) to *Xanthomonas* by affecting the iron metabolism

SUPPORTING INFORMATION LEGENDS

Figure S1 The chemical structure for Xinjunan (dioctyldiethylenetriamine).

Figure S2 Growth curves of *Xoo* cultured with Xinjunan at different concentrations.

Figure S3 Differentially expressed genes and KEGG-pathway analysis sketch.

Figure S4 qRT-PCR verification of randomly picked 6 genes.

Table S1 Determination of Xinjunan EC_{50} for 10 different *Xoo* strains

Table S2 Primers used for qRT-PCR.

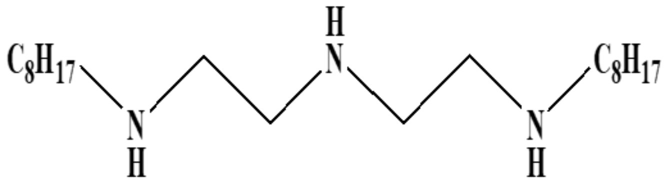

Figure S1 The chemical structure for Xinjunan (dioctyldiethylenetriamine).

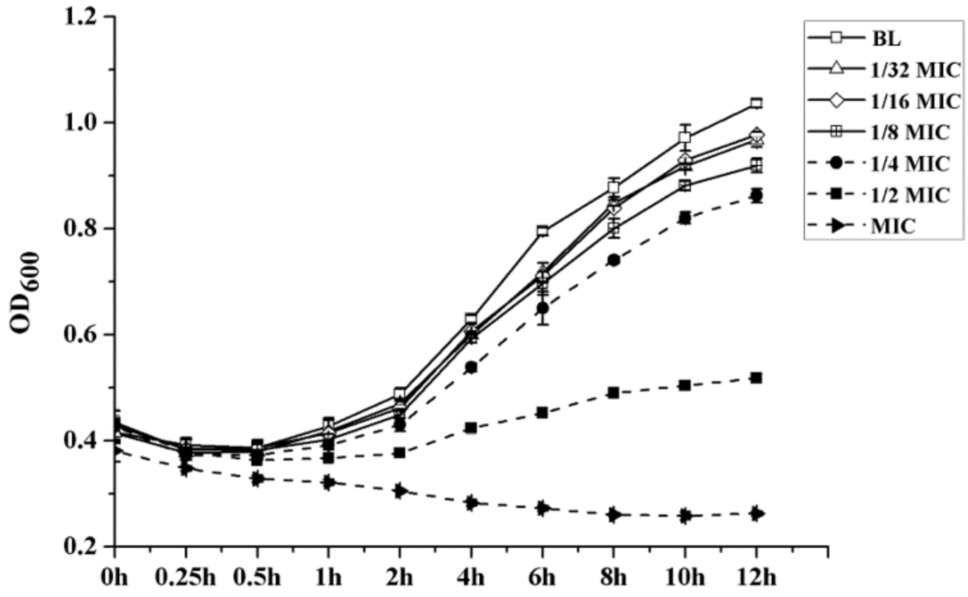

Figure S2 Growth curves of *Xoo* cultured with Xinjunan at different concentrations.

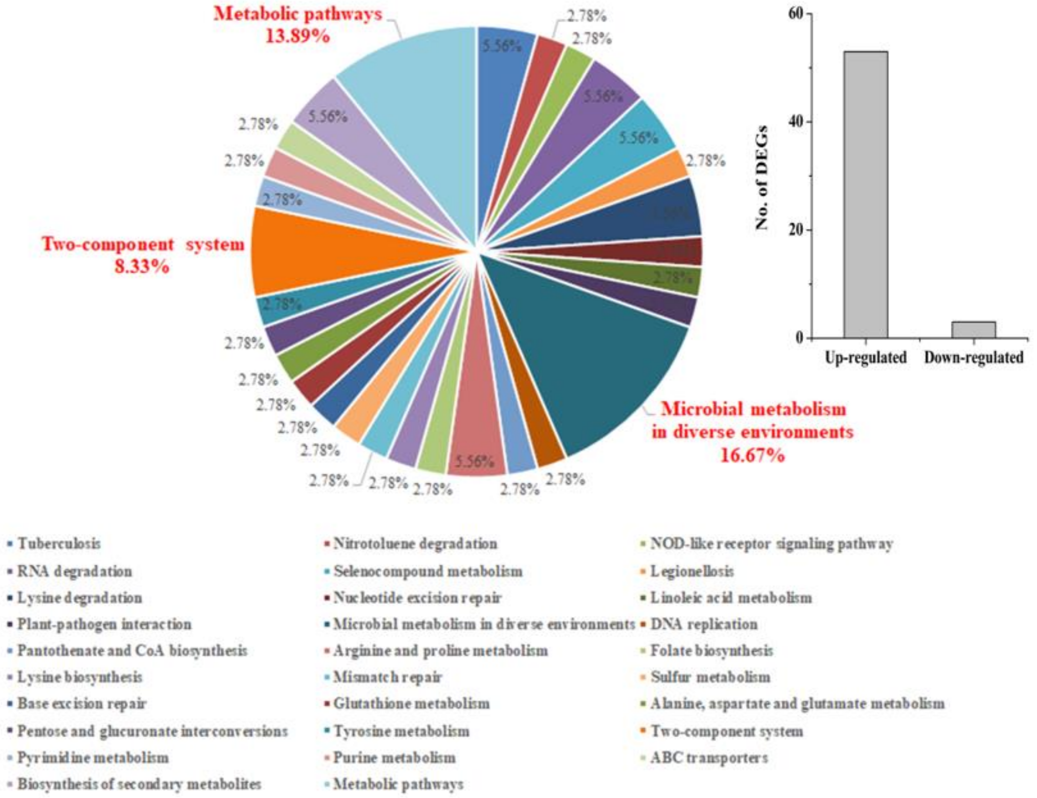

Figure S3 Differentially expressed genes and KEGG-pathway analysis sketch.

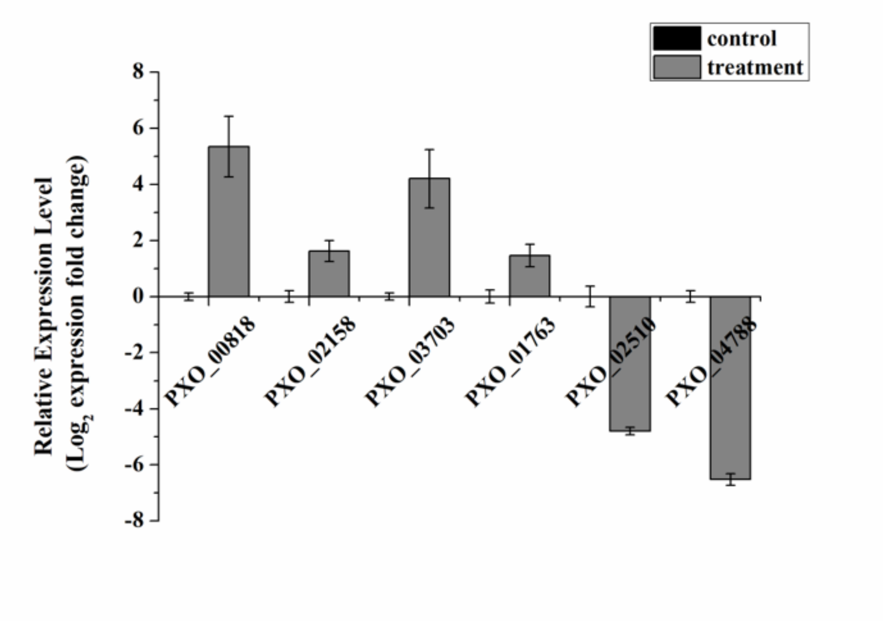

Figure S4 qRT-PCR verification of randomly picked 6 genes.

Table S1 Determination of Xinjunan EC_{50} for 10 different *Xoo* strains

**Strains**	**Regression equation**	**R^2**	**EC_{50} (mg/L)**
BYK-AAAS-01	Y=6.837+4.1146X	0.9747	0.36
BYK-AAAS-02	Y=7.4414+3.3179X	0.9208	0.18
BYK-AAAS-017	Y=6.3166+4.8724X	0.9313	0.54
BYK-AAAS-069	Y=5.6044+4.3612X	0.9440	0.73
BYK-AAAS-104	Y=4.1447+1.2188X	0.9968	5.03
BYK-AAAS-125	Y=6.5044+5.446X	0.9972	0.53
BYK-AAAS-172	Y=5.1116+1.5262X	0.9694	0.85
BYK-AAAS-1034	Y=7.1929+4.8384X	0.9569	0.35
PXO99[A]	Y=9.4995+9.359X	0.9354	0.33
R5	Y=4.9543+1.6815X	0.9610	1.06

Table S2 Primers used for qRT-PCR

**Primer name**	**Oligonucleotide sequence (5'-3')**
PvsA-F	GGCCTATTTCCACGAGCAT
PvsA-R	GCATCGCCGCCTTGTTC
PvsB-F	GCCTATCTGGTACGGCGTTAT
PvsB-R	TGTTCTGCTGATTGGCTTCC
FeoB-F	TGGTGCTGTTCGGGCTGTA
FeoB-R	GCGGATGCTCGCTCTTGT
Fur-F	CAGCACCCGATACACCG
Fur-R	AGAGCAATCACCACCACCT
16S-F	CGTATTCACCGCAGCAA
16S-R	CCAATCCCAGAAACCCTAT
